# Climate change unveils hidden microbial dangers

**DOI:** 10.1016/j.ese.2025.100544

**Published:** 2025-02-27

**Authors:** Haoxuan Yu

**Affiliations:** a Department of Civil Engineering, School of Engineering, Monash University Malaysia, Jalan Lagoon Selatan, 47500, Bandar Sunway, Selangor, Malaysia; b Monash Climate-Resilient Infrastructure Research Hub (M-CRInfra), School of Engineering, Monash University Malaysia, 47500, Bandar Sunway, Malaysia

**Keywords:** Climate change, Aquatic ecosystems, Microbial communities, Waterborne diseases, Public health

## Abstract

Climate change is driving unprecedented transformations in aquatic ecosystems, where microorganisms play a fundamental role in maintaining ecological balance and human health security. Rising water temperatures, pollution intensification, and extreme weather events are driving significant shifts in microbial community structures. These changes facilitate the proliferation of pathogenic microorganisms such as *Vibrio cholerae* and harmful algae like cyanobacteria, which thrive in warmer, nutrient-enriched environments. The resulting harmful algal blooms release potent toxins, such as microcystins, that contaminate drinking water and food supplies, leading to severe health impacts, including liver diseases and carcinogenesis. Furthermore, antibiotic resistance genes are spreading more rapidly due to climate-induced stressors, increasing the prevalence of antimicrobial-resistant pathogens and compounding the challenges for global health systems. This discussion article demonstrates that climate change influences aquatic microbial ecosystems through interconnected mechanisms, including shifts in gene transfer networks, alterations in microbial metabolism, and ecological feedback loops, ultimately increasing waterborne disease risks and antimicrobial resistance. Specific solutions are proposed, such as advancing wastewater treatment technologies to address climate-induced pollution, establishing global microbial monitoring networks leveraging remote sensing and molecular tools, and implementing early warning systems for waterborne disease outbreaks. Additionally, the discussion article emphasizes the critical role of international cooperation in funding and capacity-building efforts, particularly in developing regions with fragile infrastructures. By highlighting these pressing challenges and proposing actionable strategies, this research underscores the urgent need for integrated approaches to safeguard water resources, mitigate microbial hazards, and enhance public health resilience in an era of accelerating climate change.

## Highlights

• Climate change disrupts aquatic microbial ecosystems, driving shifts in community structures and threatening public health.
• Rising temperatures and pollution promote pathogenic microorganisms like Vibrio cholerae and cyanobacteria, causing harmful algal blooms.
• Antibiotic resistance genes spread rapidly under climate stress, escalating global health challenges from resistant pathogens.
• Solutions include advanced wastewater treatment, global monitoring networks, and early warning systems for waterborne disease outbreaks.
• International collaboration and capacity building are critical to addressing microbial hazards in regions with fragile infrastructures.

## Graphical abstract

**Figure:**
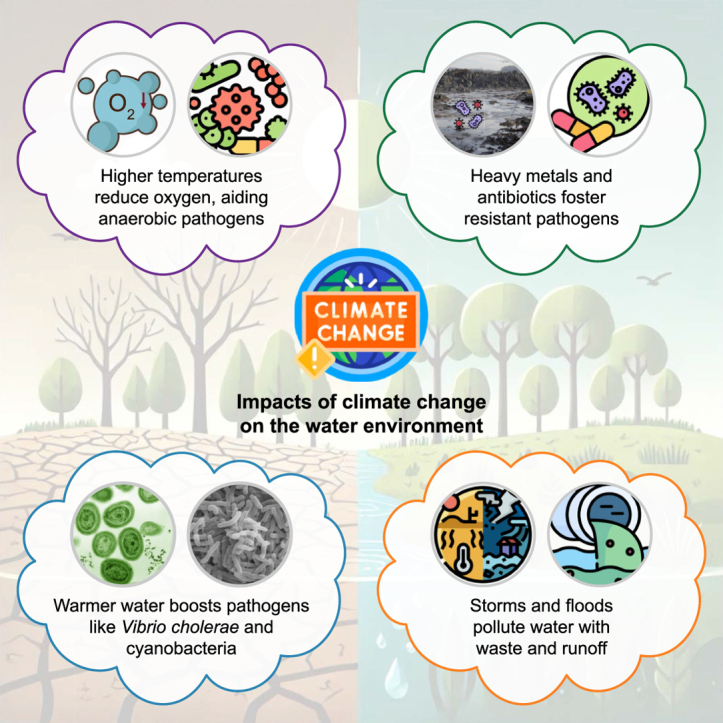

## 1 Introduction

Climate change is one of the most pressing global challenges, affecting weather patterns, ecosystems, societal structures, and human health [1,2]. Among the most vulnerable environments are aquatic ecosystems [3,4], where microorganisms are critical in maintaining ecological balance. These ecosystems are intricately linked to climate-sensitive ecological characteristics such as temperature, pH levels, and oxygen content, all altered by global warming. Microorganisms, being highly sensitive to environmental changes [5], are particularly affected by climate change. This has profound implications for various marine processes, as these organisms are integral to processes like nutrient cycling [6,7], energy flow [8,9], and pollutant breakdown [10]. Moreover, they are closely linked to the spread of pathogens and overall water safety and quality, making any disruption in their communities a potential public health concern.

The effects of climate change on aquatic ecosystems are multifaceted, with key consequences including rising water temperatures, altered precipitation patterns, and increased pollution loads. These changes disturb microbial communities and exacerbate the proliferation of pathogenic microorganisms, which thrive in warmer waters and polluted environments [11,12]. This, in turn, threatens both the ecological balance of water bodies and public health. Given these challenges, the need for a global response to safeguard water resources is particularly urgent in developing countries where water management systems and disease prevention infrastructure are less resilient. This paper will explore how climate change reshapes microbial communities in aquatic ecosystems through mechanisms such as increased water temperatures and pollution and how these changes present growing health risks that require immediate mitigation actions.

## 2 Climate Change's impact on microbial communities in aquatic ecosystems and associated health risks

**Fig. 1:**
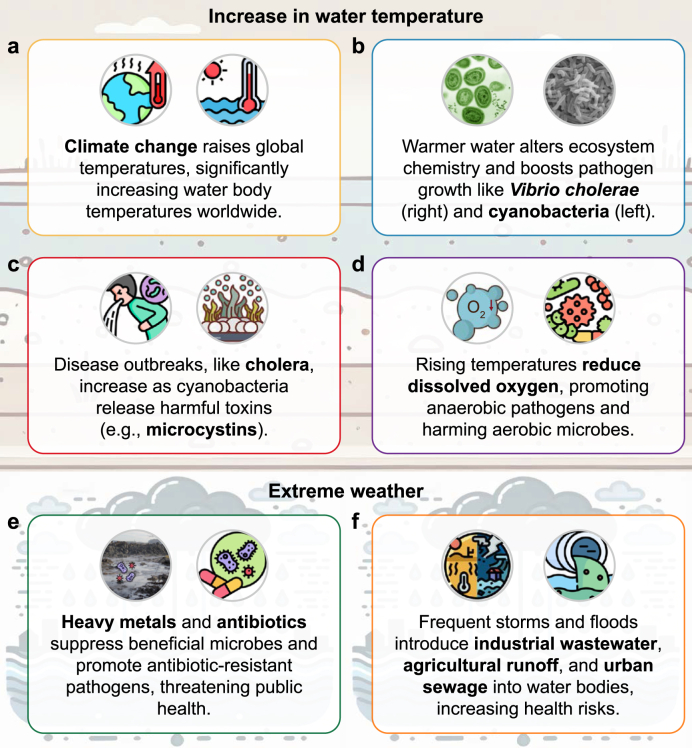
Climate change impacts aquatic microbial ecosystems. **a**, Global temperature rise's effects on water bodies; **b**, Enhanced pathogen proliferation in warmer waters; **c**, Formation and effects of harmful algal blooms; **d**, Oxygen depletion and ecosystem disruption; **e**, Industrial pollution and antimicrobial resistance; **f**, Climate-induced extreme weather impacts on water quality.

One of the most notable aspects of climate change is the steady increase in global temperatures, which directly influences water bodies, particularly freshwater lakes, rivers, and oceans. Changes in water temperature are a significant driver of microbial activity. As temperatures rise, the physical and chemical environments of aquatic systems are altered, which have profound impacts on microbial composition and function (Fig. 1a). Microorganisms are highly sensitive to temperature fluctuations, and warmer waters can lead to significant changes in their physiology, metabolic rates, and ecological functions, thereby disrupting the balance and health of entire ecosystems.

Additionally, rising temperatures contribute to ocean acidification and reduced dissolved oxygen levels [13], compounding the stress on aquatic ecosystems. Ocean acidification, caused by the uptake of atmospheric CO_2_ by seawater, lowers the pH and affects the calcification processes of marine organisms such as corals and shellfish, which are vital to the aquatic food web. Concurrently, reduced oxygen levels, a phenomenon known as ocean deoxygenation, create hypoxic zones that force aerobic microorganisms to shift to anaerobic processes [14], altering the biogeochemical cycling of carbon, nitrogen, and sulfur.

Under high-temperature conditions, many pathogenic microorganisms (e.g., *Vibrio cholerae* [15] and cyanobacteria [16,17]) can rapidly proliferate and spread (Fig. 1b). These pathogens are highly adaptable to temperature changes. In warmer waters, their metabolic rates increase dramatically, resulting in more frequent outbreaks of microbial diseases [18]. For instance, *Vibrio cholerae*, the bacterium responsible for cholera, thrives in warmer waters. It is especially prolific in tropical and subtropical regions. Research has highlighted that warmer water accelerates *Vibrio cholerae* replication and enhances toxin production, amplifying the severity of cholera outbreaks. This mechanism is tied to the thermal regulation of the bacterium's virulence genes [19]. Therefore, the incidence of cholera outbreaks is closely linked to rising water temperatures. As global warming continues, previously cooler regions may become hospitable to these pathogens. This would enable them to expand their geographical range and increase the risk of new disease outbreaks (Fig. 1c). The changes in microbial communities driven by climate change pose significant public health concerns, with the risk of waterborne diseases rising alongside the spread of pathogens.

Moreover, rising water temperatures can trigger the widespread growth of harmful algae, particularly cyanobacteria, which contribute to harmful algal blooms (HABs) [20,21]. These blooms often occur in nutrient-rich waters and proliferate under warm conditions. Rising temperatures enhance the stratification of water bodies, limiting vertical mixing and creating warm, stable layers near the surface that are ideal for cyanobacteria growth. Additionally, elevated temperatures accelerate the metabolic rates of these algae, boosting their ability to outcompete other phytoplankton species and thereby allowing them to dominate aquatic ecosystems [22]. HABs severely pollute water sources, disrupting the ecological balance of aquatic systems and releasing toxins, such as microcystins (Fig. 1c). These toxins can have severe health implications for humans [20]. Microcystins are potent hepatotoxins produced during cyanobacteria blooms. These toxins inhibit protein phosphatases 1 and 2A, critical enzymes in cellular signaling. In humans, this leads to oxidative stress and hepatocyte apoptosis, which can progress to liver diseases and even hepatocellular carcinoma [23,24]. In recent years, the frequency of cyanobacteria blooms has increased in freshwater lakes and rivers, particularly in nutrient-enriched environments. Climate change has exacerbated this issue by intensifying extreme weather events, such as heavy rainfall, which increases nutrient runoff from agricultural lands into water bodies. This nutrient loading, combined with rising temperatures, creates a feedback loop that further encourages HAB proliferation [25]. The consequences of these blooms extend beyond pollution—they also deplete dissolved oxygen in the water [26]. Without adequate oxygen, fish and other aquatic organisms die off, further disrupting the ecosystem (Fig. 1d). When these toxins enter drinking water supplies or the food chain, they can lead to various health issues, including gastrointestinal discomfort, liver damage, and neurotoxicity [27].

Beyond rising temperatures, extreme weather events, such as heavy rainfall and flooding, are becoming critical factors in water pollution levels. These events lead to a surge in surface runoff, which carries visible debris, dissolved chemicals, pathogens, and excess nutrients into aquatic ecosystems [28]. Heavy rainfall intensifies soil erosion, introducing sediments that further disturb the light penetration and temperature stability of water bodies. This cascading effect can dramatically alter microbial activity and ecological interactions in affected water systems. Changing precipitation patterns and more frequent extreme weather events can also introduce significant quantities of pollutants from industrial wastewater, agricultural runoff, and urban sewage into water bodies (Fig. 1e). Agricultural runoff, for example, is rich in nitrogen and phosphorus. During heavy rainfall or flooding, more fertilizers are washed into rivers and lakes, dramatically increasing nutrient levels [29]. This eutrophication further fuels the growth of harmful algae, especially cyanobacteria. Moreover, the degradation of algal biomass consumes large amounts of oxygen, leading to hypoxic or even anoxic conditions that devastate fish populations and other aerobic aquatic life [30]. The resultant nutrient overload disrupts the ecological balance of water systems and produces large amounts of toxic algal blooms, as mentioned above.

Extreme weather events also lead to increased contamination by industrial pollutants such as heavy metals, organic chemicals, and antibiotics [31,32]. For instance, flooding often overwhelms wastewater treatment systems, causing untreated or partially treated sewage laden with industrial and pharmaceutical waste to spill into nearby water bodies. This leads to a significant rise in pollutant concentrations, including antibiotic residues, heavy metals, and persistent organic pollutants, which pose severe ecological and health risks [33]. These pollutants can selectively affect microbial communities, inhibiting beneficial microbes and promoting the growth of antibiotic-resistant pathogens (Fig. 1f). For example, the accumulation of antibiotic residues in water can drive the spread of resistance genes within microbial communities, increasing resistant pathogens. These residues act as selective pressures, favoring the survival and multiplication of resistant bacterial strains over susceptible ones. Heavy metals such as mercury and cadmium also play a role by co-selecting for resistance genes through shared genetic platforms like plasmids [34]. Once these pathogens enter the human body, they can cause serious infections that are difficult to treat with conventional antibiotics. As climate change accelerates water pollution, these antibiotic-resistant pathogens proliferate and spread, further endangering public health and leading to potential crises.

When urban and rural wastewater treatment systems are overwhelmed [35,36], untreated sewage can be discharged into natural water bodies. As discussed, flooding can inundate treatment facilities, causing system failures that result in raw sewage spilling into rivers and lakes. This disrupts aquatic ecosystems, creating ideal conditions for waterborne diseases to spread rapidly. In Mozambique, severe flooding events have been linked to sharp increases in cholera cases, as contaminated water and damaged sanitation infrastructure heighten health risks [37]. These untreated effluents contain large quantities of pathogens, including bacteria such as *Escherichia coli* and *Salmonella*, as well as parasite eggs [38,39].

Furthermore, the destruction of sanitation infrastructure during such events often leaves communities without access to clean water, compounding the risks of outbreaks. In Somalia, recurrent flooding has destroyed critical water and sanitation infrastructure, leading to repeated cholera epidemics. These events underscore the fragility of public health systems in regions facing climate-induced disasters [40]. These contaminants in drinking or irrigation water supplies pose direct health risks, particularly in areas with inadequate sanitation infrastructure. Outbreaks of diseases such as cholera and dysentery are rising in these regions, placing additional strain on already fragile public health systems.

The United Nations has emphasized the urgent need for resilient water and sanitation systems capable of withstanding the impacts of extreme weather events [41]. Strengthening these systems is essential to prevent the collapse of sanitation services during disasters, which can lead to severe public health crises. This requires international cooperation, funding, and capacity building, particularly in developing countries where infrastructure is most vulnerable to climate shocks. Many developing areas are already grappling with climate-induced water pollution, an urgent threat to global public health.

Therefore, climate change significantly exacerbates water pollution and its impact on microbial communities in ways that extend far beyond the ecological sphere. Rising water temperatures are altering microbial dynamics and promoting the proliferation of harmful algae and pathogens while extreme weather events such as floods overwhelm wastewater systems, introducing pollutants and untreated sewage into natural water bodies. These consequences of climate change intensify the risk of waterborne diseases and create hotspots for antibiotic-resistant pathogens. These effects destabilize aquatic ecosystems and increase the speed and extent of pathogen spread, directly threatening human health. Without effective measures to control water pollution and address the impacts of climate change on aquatic ecosystems, the future may see more frequent outbreaks of water-related diseases, posing a serious threat to global public health. Addressing water pollution and protecting aquatic ecosystems in the face of climate change are major challenges for nations worldwide, requiring multilateral cooperation and technological innovation to ensure water safety and ecological health.

## 3 Mechanisms of microbial community evolution in aquatic ecosystems under climate change

Climate change profoundly impacts microbial communities' structure and function in aquatic ecosystems. Previous sections discussed how rising temperatures and extreme weather events alter aquatic environments, leading to pathogen proliferation and HABs. In the face of these changes, microbial communities are undergoing complex evolutionary processes. Rising water temperatures modify microbial physiological metabolism and affect competition among functional groups. Meanwhile, environmental stress reshapes gene transfer networks among microorganisms, accelerating the spread of resistance genes. These changes affect both ecosystem health and public health security.

This section explores two key questions: how climate change influences antibiotic resistance gene (ARG) transfer within microbial communities and whether rising water temperatures universally promote pathogen community growth. The first concerns antibiotic resistance transmission mechanisms, while the second concerns water safety. By analyzing community evolution patterns under environmental stress, we can better understand climate change's profound impact on aquatic ecosystems and provide scientific evidence for predicting and addressing potential risks.

### 3.1 Selection pressure and transmission mechanisms of ARGs under climate change

**Fig. 2:**
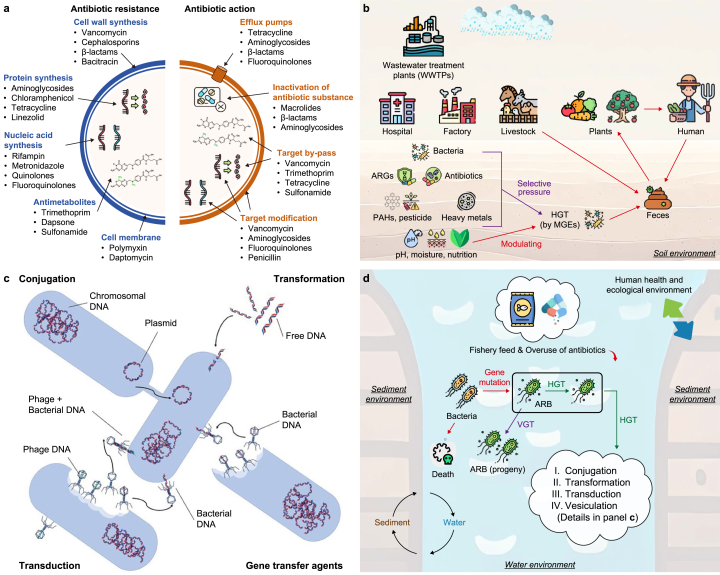
Mechanisms of antibiotic resistance gene (ARG) transmission under climate change: a, Antibiotic targets and bacterial resistance mechanisms (Adapted from Ref. [43]. Copyright CC BY 4.0); b, Environmental factors affecting antibiotic resistance gene (ARG) transmission (Adapted from Ref. [45]. Copyright CC BY 4.0); c, Horizontal gene transfer pathways (Adapted from Ref. [44]. Copyright CC BY 4.0); d, Antibiotic resistance gene (ARG) transmission network in aquaculture systems (Adapted from Ref. [46]. Copyright CC BY 4.0). ARGs: antibiotic resistance genes; WWTPs: wastewater treatment plants; HGT: horizontal gene transfer; MGEs: mobile genetic elements; PAHs: polycyclic aromatic hydrocarbons; DNA: deoxyribonucleic acid; ARB: antibiotic resistant bacteria; VGT: vertical gene transfer.

Climate change influences ARG transmission patterns in aquatic ecosystems through multiple pathways, including temperature rise, extreme weather events, and pollutant inputs. These environmental changes affect microbial physiology and accelerate ARGs spread by alterations in community structure and gene transfer efficiency [42]. Antibiotics and resistance mechanisms form complex interaction networks involving multiple targets, including cell wall synthesis, protein synthesis, and nucleic acid synthesis (Fig. 2a) [43,44]. These targets and their corresponding resistance mechanisms show varying responses when environmental temperatures rise.

As the most direct environmental pressure, temperature increase affects ARG transmission across multiple levels. When water temperatures exceed 33–35 °C, water physicochemical properties and bacterial physiological states are significantly altered [47,48]. Fig. 2b illustrates how environmental factors (temperature, pH, and nutrients) regulate ARG transmission through selection pressure. Under high temperatures, increased bacterial membrane fluidity and permeability promote plasmid-mediated horizontal gene transfer (HGT), while activated save our ship (SOS) response systems enhance transposon activity, providing more genetic vectors for ARG transfer [49,50].

ARG transmission primarily occurs through HGT. Fig. 2c shows three main transfer mechanisms: conjugation (direct cell contact), transformation (exogenous deoxyribonucleic acid [DNA] uptake), and transduction (phage-mediated transfer). Higher temperatures significantly enhance these transfer mechanisms’ efficiency. Particularly for conjugation, elevated temperatures promote membrane fluidity and increase plasmid transfer frequency while enhancing bacterial competence probability [51]. Fig. 2d further demonstrates how gene mutation, vertical gene transfer (VGT), and HGT form complete ARG transmission networks in environments like aquaculture.

Extreme weather events and pollutant inputs constitute another crucial factor that facilitates ARG transmission. Fig. 2b details how human facilities (wastewater treatment plants, pharmaceutical factories, and hospitals) release ARGs through various pathways. Increased surface runoff from storms and floods carries antibiotic residues and heavy metal pollutants. These pollutants influence ARG enrichment through co-selection mechanisms (Fig. 2b). This phenomenon is significant because heavy metals and ARGs often co-locate on the same mobile genetic elements.

As such, sewage treatment systems are facing new challenges under climate change. Antibiotic resistance involves multiple molecular mechanisms that exhibit different activities under high temperatures (Fig. 2a). Temperature increases affect treatment efficiency, particularly in biological treatment units. Extreme weather events may lead to facility overload, making them crucial nodes for ARG transmission. Here, ARGs from different sources, antibiotic residues, and heavy metals interact to form complex selection pressure networks.

Climate change also reshapes ARG transmission networks by influencing microbial adaptive evolution and community distribution. Fig. 2d shows how gene mutation and horizontal transfer play crucial roles in bacterial adaptation to environmental stress. Continuous temperature stress increases mutation rates and affects DNA repair system efficiency, accelerating resistance gene evolution. Fig. 2b shows how environmental factors (pH, moisture, and nutrition) change microbial community structure and function, affecting ARG spatial distribution patterns.

These complex influence mechanisms interweave to affect molecular-level resistance mechanisms (Fig. 2a), community-level gene transfer (Fig. 2c), and ecosystem-level environmental impacts (Fig. 2b–d), collectively shaping ARG transmission dynamics under climate change. Understanding these multilevel mechanisms is crucial for predicting and controlling antibiotic resistance spread and provides scientific bases for prevention strategies. Under intensifying global warming, we need more systematic monitoring and assessment of climate change impacts on ARG transmission to establish multilevel prevention systems to maintain aquatic ecosystem health and public health security.

### 3.2 Temperature rise: comprehensive impacts and dynamic mechanisms on pathogen populations

**Fig. 3:**
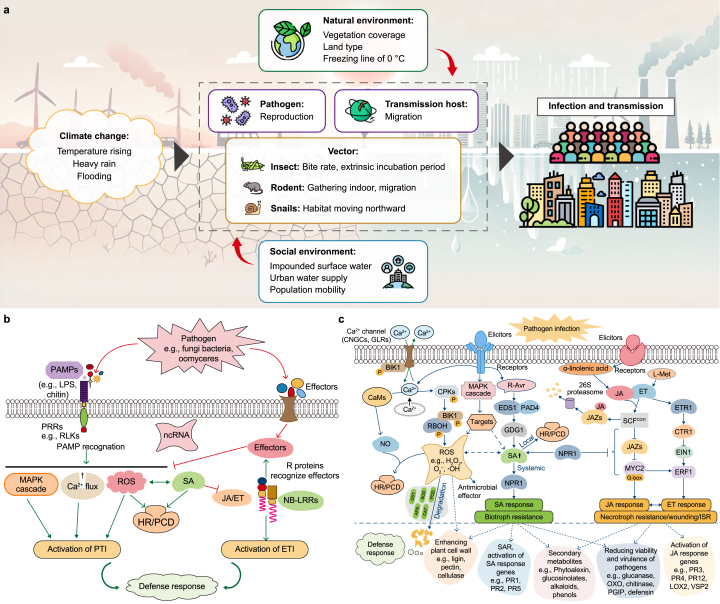
Temperature effects on pathogen population dynamics: a, Climate change impacts on pathogen environments (Adapted from Ref. [52]. Copyright CC BY 4.0); b, Molecular signaling in pathogen infection processes (Adapted from Ref. [55]. Copyright CC BY 4.0); c, Temperature-mediated pathogen–host interactions (Adapted from Ref. [55]. Copyright CC BY 4.0).

As a direct consequence of climate change, temperature rise exerts broad and profound effects on pathogen populations in aquatic ecosystems. According to a study by Wu et al. (see Fig. 3a) [52], temperature elevation can directly promote the growth and reproduction of most pathogens. This effect is primarily attributed to the shortening of viral incubation periods and reproduction cycles, the acceleration of pathogen lifecycles, and their significantly improved survival rates. These changes are particularly evident in pathogens that have adapted to higher temperatures, such as certain *Vibrio* species and *Pseudomonas aeruginosa*, which demonstrate enhanced proliferation capabilities after water temperature increases [53,54]. However, rising temperatures do not universally benefit all pathogens. When temperatures exceed certain thresholds (e.g., consistently remaining above 37 °C), reproduction may be inhibited by mechanisms such as water evaporation, oxygen reduction, or nutrient dilution, especially for species with low heat tolerance [52].

PAMPs: pathogen-associated molecular patterns; LPS: lipopolysaccharide; PRRs: pattern recognition receptors; RLKs: receptor-like kinases; ncRNA: non-coding RNA; MAPK: mitogen-activated protein kinase; Ca^2+^: calcium ion; ROS: reactive oxygen species; SA: salicylic acid; HR/PCD: hypersensitive response/programmed cell death; JA/ET: jasmonic acid/ethylene; NB-LRRs: nucleotide-binding leucine-rich repeats; PTI: PAMP-triggered immunity; ETI: effector-triggered immunity; CNGCs: cyclic nucleotide-gated channels; GLRs: glutamate receptor-like; BIK1: botrytis-induced kinase 1; CaMs: calmodulins; CPKs: calcium-dependent protein kinases; R-Avr: resistance-avirulence; EDS1: enhanced disease susceptibility 1; PAD4: phytoalexin deficient 4; JA: jasmonic acid; ET: ethylene; JAZs: jasmonate ZIM-domain proteins; SCF: Skp1-Cullin-F-box; ETR1: ethylene response 1; CTR1: constitutive triple response 1; EIN1: ethylene insensitive 1; MYC2: MYC2 transcription factor; ERF1: ethylene response factor 1; NPR1: non-expressor of pathogenesis-related genes 1; RBOH: respiratory burst oxidase homolog; NO: nitric oxide; GDG1: G-type lectin receptor-like kinase 1; PR1, PR2, PR5: pathogenesis-related proteins 1, 2, and 5; PR3, PR4, PR12: pathogenesis-related proteins 3, 4, and 12; LOX2: lipoxygenase 2; VSP2: vegetative storage protein 2; SAR: systemic acquired resistance; OXO: oxalate oxidase; PGIP: polygalacturonase-inhibiting protein; L-Met: L-Methionine; ISR: induced systemic resistance.

Behind this widespread impact of temperature rise on pathogen populations lies a complex set of dynamic mechanisms that extend beyond direct physiological effects. Referencing another study by Ding et al. (Fig. 3b) [55], temperature changes trigger a series of signal cascade reactions, paralleling plant immune response networks. These reactions include interconnected pathways such as calcium ion signaling, mitogen-activated protein kinase (MAPK) cascades, and reactive oxygen signaling [56]. These signal networks directly affect pathogen growth rates and toxicity expression by regulating cellular metabolic pathways. For instance, under high-temperature conditions, the MAPK signaling pathway can be activated, potentially enhancing the toxin secretion capabilities of certain pathogens and thereby further intensifying their threat to ecological systems and public health [57,58].

At the ecological level, temperature rise indirectly influences pathogen population dynamics by altering habitats and resource availability. Temperature elevation may change vegetation coverage and land use types, affecting pathogen and vector habitats (Fig. 3a). For example, habitat loss caused by temperature increases may push certain wildlife species into human settlement areas, intensifying pathogen transmission opportunities [59]. Additionally, temperature rise can compromise urban water supply systems, where biofilm formation under high-temperature conditions provides ideal shelters for pathogens [60]. These disruptions to ecosystem balance significantly alter the reproduction, survival, and transmission patterns of pathogens, vectors, and intermediate hosts, amplifying their ecological and public health impacts.

Temperature rise also accelerates nutrient cycling, profoundly influencing resource availability for pathogen populations. Mechanisms similar to those in plant defense signal transduction networks are activated under high-temperature conditions (Fig. 3c), facilitating the rapid cycling of essential elements like carbon, nitrogen, and phosphorus [61]. In eutrophic water bodies, such as during cyanobacterial blooms, pathogens often attach to algal surfaces to obtain abundant nutrients and significantly increase their reproduction rates [62]. This synergistic relationship between temperature rise and eutrophication alters pathogen population structures, creating feedback loops that intensify ecological imbalances.

Nevertheless, the impact of temperature rise on pathogens is not uniformly promotive. Resource competition and population dynamics and regulation introduce complexity into these interactions. For instance, high temperatures may decrease dissolved oxygen content, limiting aerobic pathogens while favoring anaerobic species [63]. Simultaneously, competition among pathogens intensifies, with certain species secreting metabolic inhibitory substances (e.g., antimicrobial peptides) to suppress rivals [64].

The interaction between temperature rise and pathogen populations is thus a multifaceted ecological process involving direct physiological changes and intricate ecosystem-level dynamics. Different types of pathogens (e.g., insect-transmitted or rodent-transmitted) exhibit varying responses to temperature changes depending on their ecological niches and adaptability. This variability underscores the importance of context-specific analyses that account for regional and environmental differences.

In summary, temperature rise profoundly influences pathogen population growth and transmission through interconnected pathways at the molecular, population, and ecosystem levels. Understanding these mechanisms offers valuable insights for predicting pathogen behavior under climate change scenarios and developing targeted public health strategies to mitigate waterborne disease risks.

## 4 Conclusion

Climate change alters aquatic microbial ecosystems through rising temperatures, pollution escalations, and increasingly frequent extreme weather events. Consequently, these environmental shifts create unprecedented challenges for ecological balance and global public health.

Water bodies are becoming fertile breeding grounds for pathogenic microorganisms in this rapidly changing landscape. Warm, nutrient-rich waters enable *Vibrio cholerae* to replicate more aggressively and produce heightened toxin levels, escalating the potential for severe cholera outbreaks. Moreover, cyanobacterial blooms, driven by eutrophication and temperature increases, release potent microcystins that pose significant threats to aquatic life and human health, potentially causing liver damage and increasing hepatocellular carcinoma risks.

The alarming spread of ARGs in aquatic systems further complicates the situation. Environmental pollution and extreme weather events are accelerating the proliferation of these genes. The result is resistant pathogens that challenge existing medical interventions and undermine public health infrastructures.

Confronting these intricate challenges requires a comprehensive, innovative approach. Advanced wastewater treatment technologies must be developed to eliminate antibiotic residues, heavy metals, and pollutants that drive microbial mutations. Simultaneously, global monitoring networks employing remote sensing and molecular biology techniques can offer real-time detection of emerging microbial threats, especially in resource-limited regions.

Ecosystem restoration emerges as a critical strategy in this complex scenario. By reducing nutrient loading, implementing stringent pollution controls, and promoting sustainable agricultural practices, we can mitigate HABs and restore ecological equilibrium. Community education and early warning systems will further empower local populations to adopt proactive water conservation practices.

International collaboration will be the linchpin of these efforts. Multilateral agreements and dedicated funding mechanisms can support research, enable data sharing, and provide targeted resources for vulnerable regions. Such collaborative frameworks can help develop more adaptive and resilient strategies to address climate-induced microbial challenges.

As environmental transformations accelerate, interdisciplinary cooperation has become imperative. Researchers must prioritize understanding regional variations in microbial responses to environmental stressors to develop interventions considering local ecological and socioeconomic contexts. We can create more nuanced and effective approaches to these complex challenges by bridging disciplines and perspectives.

Ultimately, mitigating these threats demands sustained commitment, innovative thinking, and unprecedented global cooperation. By comprehensively addressing the intricate relationships between climate change, microbial ecosystems, and public health, we can forge a path toward a more resilient and sustainable future, directly supporting critical Sustainable Development Goals.

## Copyright notice

**Graphical Abstract** and Fig. 1 contains various graphic elements sourced from free-license resources by Freepik, Vectors Tank, surang, Flat Icons, Eucalyp, Creative Stall Premium, pongsakornRed, and Iconjam. In particular, the *Vibrio cholerae* illustration in Fig. 1b is Free to use (Public Domain), and Cyanobacteria (Blue-green algae) is licensed under CC0 1.0 Universal. The Heavy metals illustration in Fig. 1e is licensed under CC BY 3.0. All elements in Fig. 2, Fig. 3 are adapted from original works by the author or licensed under the Creative Commons Attribution International License (CC BY). All non-original images are properly credited and cited according to their respective licensing requirements.

## Declaration of competing interest

The authors declare that they have no known competing financial interests or personal relationships that could have appeared to influence the work reported in this paper.
